# Erratum to: Randomized comparison of atrioventricular node re-entry tachycardia and atrial flutter catheter ablation with and without fluoroscopic guidance: ZeroFluoro study

**DOI:** 10.1093/europace/euad311

**Published:** 2023-10-25

This is an erratum to: Frantisek Lehar, Nándor Szegedi, Jakub Hejc, Jiri Jez, Filip Soucek, Tomas Kulik, Anna Siruckova, Zoltan Sallo, Klaudia Vivien Nagy, Bela Merkely, László Geller, Zdeněk Starek, Randomized comparison of atrioventricular node re-entry tachycardia and atrial flutter catheter ablation with and without fluoroscopic guidance: ZeroFluoro study, *EP Europace*, Volume 24, Issue 10, October 2022, Pages 1636–1644, https://doi.org/10.1093/europace/euac049

In the originally published version of this manuscript, mark-up and statements of equal first authorship were missed. The authorship list should read:

“Frantisek Lehar^1,2,†^, Nándor Szegedi^3,†^, Jakub Hejc^1^, Jiri Jez^1,2^, Filip Soucek^1,2^, Tomas Kulik^1,2^, Anna Siruckova^1,2^, Zoltan Sallo^3^, Klaudia Vivien Nagy^3^, Bela Merkely^3^, László Geller^3^, and Zdeněk Starek^1,2^*” instead of “Frantisek Lehar^1,2^, Nándor Szegedi^3^, Jakub Hejc^1^, Jiri Jez^1,2^, Filip Soucek^1,2^, Tomas Kulik^1,2^, Anna Siruckova^1,2^, Zoltan Sallo^3^, Klaudia Vivien Nagy^3^, Bela Merkely^3^, László Geller^3^, and Zdeněk Starek^1,2^*”. There should be a corresponding additional statement in the PDF: “^†^Equal first authors.” and within the **Author Notes** online: “Frantisek Lehar and Nándor Szegedi share equal first authorship.”

These lacunae have been outlined only in this erratum to preserve the version of record.

